# Nanodiamond enhanced mechanical and biological properties of extrudable gelatin hydrogel cross-linked with tannic acid and ferrous sulphate

**DOI:** 10.1186/s40824-022-00285-3

**Published:** 2022-07-30

**Authors:** Amitava Bhattacharyya, V. N. Karthikai Priya, Ji-hyeon Kim, Mst Rita Khatun, R. Nagarajan, Insup Noh

**Affiliations:** 1 Department of Chemical and Biomolecular Engineering, Seoul National University of Science and Technology, Seoul, 01811 Republic of Korea; 2 Convergence Institute of Biomedical Engineering and Biomaterials, Seoul National University of Science and Technology, Seoul, 01811 Republic of Korea; 3 Functional, Innovative and Smart Textiles, PSG Institute of Advanced Studies, Coimbatore, 641004 India

**Keywords:** Hydrogel, Gelatin, Tannic acid, Ferrous sulphate, Nanodiamond

## Abstract

**Background:**

The requirements for cell-encapsulated injectable and bioprintable hydrogels are extrusion ability, cell supportive micro-environment and reasonable post-printing stability for the acclimatization of the cells in the target site. Detonation nanodiamond (ND) has shown its potential to improve the mechanical and biological properties of such hydrogels. Enhancing the performance properties of natural biopolymer gelatin-based hydrogels can widen their biomedical application possibilities to various areas including drug delivery, tissue engineering and 3D bioprinting.

**Method:**

In this study, natural cross-linker tannic acid (TA) is used along with ferrous sulphate (FS) to optimize the swelling and disintegration of extrudable and 3D printable gelatin hydrogels. The amounts of TA and FS are restricted to improve the extrusion ability of the gels in 3D printing. Further, ND particles (detonation type) are dispersed using twin screw extrusion technology to study their effect on mechanical and biological properties of the 3D printing hydrogel.

**Results:**

The improved dispersion of ND particles helps to improve compressive strength almost ten times and dynamic modulus three times using 40 mg ND (2% w/w of gelatin). The surface-functional groups of detonation ND also contributed for such improvement in mechanical properties due to higher interaction with the hydrogel matrix. The stability of the hydrogels in water was also improved to 7 days. Four times improvement of the cell growth and proliferation was observed in ND based hydrogel.

**Conclusion:**

The cell-supportive nature of these moderately stable and extrudable ND dispersed gelatin hydrogels makes them a good candidate for short term regenerative applications of cell-encapsulated injectable hydrogels with better mechanical properties.

**Supplementary Information:**

The online version contains supplementary material available at 10.1186/s40824-022-00285-3.

## Introduction

Hydrogel, a water-swollen, three dimensional (3D) cross-linked polymeric network, has been employed as a composite matrix in 3D bioprinting and a tissue engineering scaffold [1]. Hydrogel may be chemically stable, or they may disintegrate, degrade and dissolve depending on the nature of functional groups present in their structure [2]. Due to their high-water content, network porosity, and soft consistency, they closely resemble natural live tissue than any other class of biomaterials [3]. Recently they are widely used as a major bioink component in 3D printing or bioprinting due to their biocompatibility [4]. They have also a great potential for drug delivery via different administration routes like oral, nasal, buccal, rectal, vaginal, eye, injection, and others [5].

One potential hydrogel-forming biopolymer is gelatin, the denaturized collagen, among many other hydrogel materials. It is extensively used in pharmaceutical, food, medical and tissue engineering applications. The gel formed by physical crosslinking of gelatin is not structurally stable and dissolve rapidly at elevated temperature [6]. Hence, chemical crosslinking is recommended to overcome its poor mechanical and structural retention properties in its gel form uses [7]. Tannic acid (TA), a natural nontoxic phenolic crosslinker, is found to be suitable for improving the mechanical and antibacterial properties of gelatin/TA film [8]. TA has many phenol groups that can bind with proteins through both hydrophobic interactions and hydrogen bonding [4, 5]. Interfacial interaction between TA and gelatin takes place between the amino group of gelatin and hydroxyl group of TA due to hydrogen bonding [6]. The oxidation of polyphenol groups of TA using sodium periodate was found to improve the self-healing and adhesive properties of gelatin hydrogels [9].

Furthermore, hydrogels formed by proteins have limited use in medical fields due to poor physical properties, especially in 3D printable forms where post-printing fidelity and mechanical properties are extremely important. Hence, double crosslinking is introduced by researchers for better network formation [10]. TA forms metal complex with iron [11] which has the potential to form a better cross-linked network in gelatin/TA system. Among the metal ion cross-linked hydrogels, iron is reported as a crosslinker by several researchers in recent years [12]. It is an essential body mineral for growth and development. Ge et al. (2019) prepared double cross-linked gelatin hydrogels with improved mechanical properties using oxidized TA and ferrous sulphate (FeSO4, FS) [7]. FS is an iron salt that absorbs well in human body, hence, is used to treat iron deficiency anaemia.

In addition to those matrix improvement methods, nano-biomaterials are often used to strengthen the polymer matrix of hydrogels in 3D bioprinting. The 3D printed or bioprinted structures reinforced with suitable nanoparticles not only improve their mechanical and biological properties, but also reduce the rate of scaffold degradation [13]. Among the carbon nanomaterials, nanodiamond (ND) has the potential to become an outstanding biomaterial due to its higher biocompatibility. The nanosized carbon building blocks in ND help it to become a successful carrier for biomedical and tissue engineering application. NDs have long time fluorescence and are photostable over longer periods. This has the possibility of utilizing ND for imaging and early diagnosis [14]. Other applications include biosensors, transducers in chemical sensing, and semiconductors (boron-doped ND). ND-based drug delivery is under study for tumor therapies and gene delivery. The surface of ND can be functionalized with various functional groups like ether-, hydroxyl-, carbonyl-, carboxyl-, and amino- groups. Among the various processes to synthesize ND, detonation process is most advantageous as it offers various functional groups on ND surface during the synthesis process [15]. Hydrophobic drugs can be adsorbed on the surface of such NDs, which enhances their dispersibility in water. Detonation ND is easy to prepare in large scale and have small size distribution. Though it has good water-dispersibility, the presence of different functional groups and SP^2^ carbon affects the stability of ND and often leads to agglomeration in dispersing media [14].

Nanocomposites with ND and biopolymers have outstanding mechanical properties and excellent chemical resistance. Issues like aggregation, degradation by-products, control of surface chemistry and poor cell affinity need to be addressed for its successful application in tissue engineering [16]. A nanohybrid system created using gelatin, chitosan and ND exhibited better control over release kinetics than conventional hydrogel. The injectable nanocomposite hydrogel showed complete strain recovery and maintained a steady state release of exogenous human vascular endothelial growth factor (VEGF) for wound healing applications. The in vitro study of apoptotic gene expressions and anti-inflammatory interleukin confirmed the biocompatibility of this nanocomposite hydrogel [17]. NDs are used to improve the mechanical properties of gelatin-methacrylamide (GelMA: a photo cross-linkable hydrogel) by stiffening the gel network. The release of an osteogenic differentiating drug (dexamethasone) to human adipose derive stem cells was modulated using ND for increased alkaline phosphatase activity and calcium deposition [18].

In this study, the possibility of using TA and FS as biocompatible cross-linking agents for preparation of extrudable and stable gelatin gel is studied for 3D printing applications. The effects of ND on mechanical and biological properties of such gels are also examined. Homogeneous distribution of ND in the gelatin hydrogel is ensured using a twin screw extrusion (TSE) head.

## Experimental

### Method

2 g of extra pure grade gelatin (Himedia, India, Jelly/Gel (Bloom) strength: 150—250 g, and pH (1% in water at 55 °C): 3.80—7.60) was dissolved in 8 ml of deionized (DI) water at 60 ºC in a magnetic stirrer and cooled. Tannic acid (Biochemika reagents, India) was dissolved in 2 ml of DI water, separately. The tannic acid (TA) solution was mixed with the gelatin solution and ferrous sulphate (FS, Loba Chemie, India) was added with stirring for 15 min. Hence, the final concentration of gelatin in the hydrogel was 20% w/v. The mixture was kept undisturbed for 1 h. Initially different combinations were tried and examined for good extrusion ability and stability. With different amount of TA and FS, the gelatin gels were prepared using the same procedure and checked for good extrusion ability, disintegration and swelling in water for 5 days. With the results, the amounts of TA and FS were optimized to 0.05 g and 0.02 g, respectively, for nanocomposite gel preparation.

**Fig. 1:**
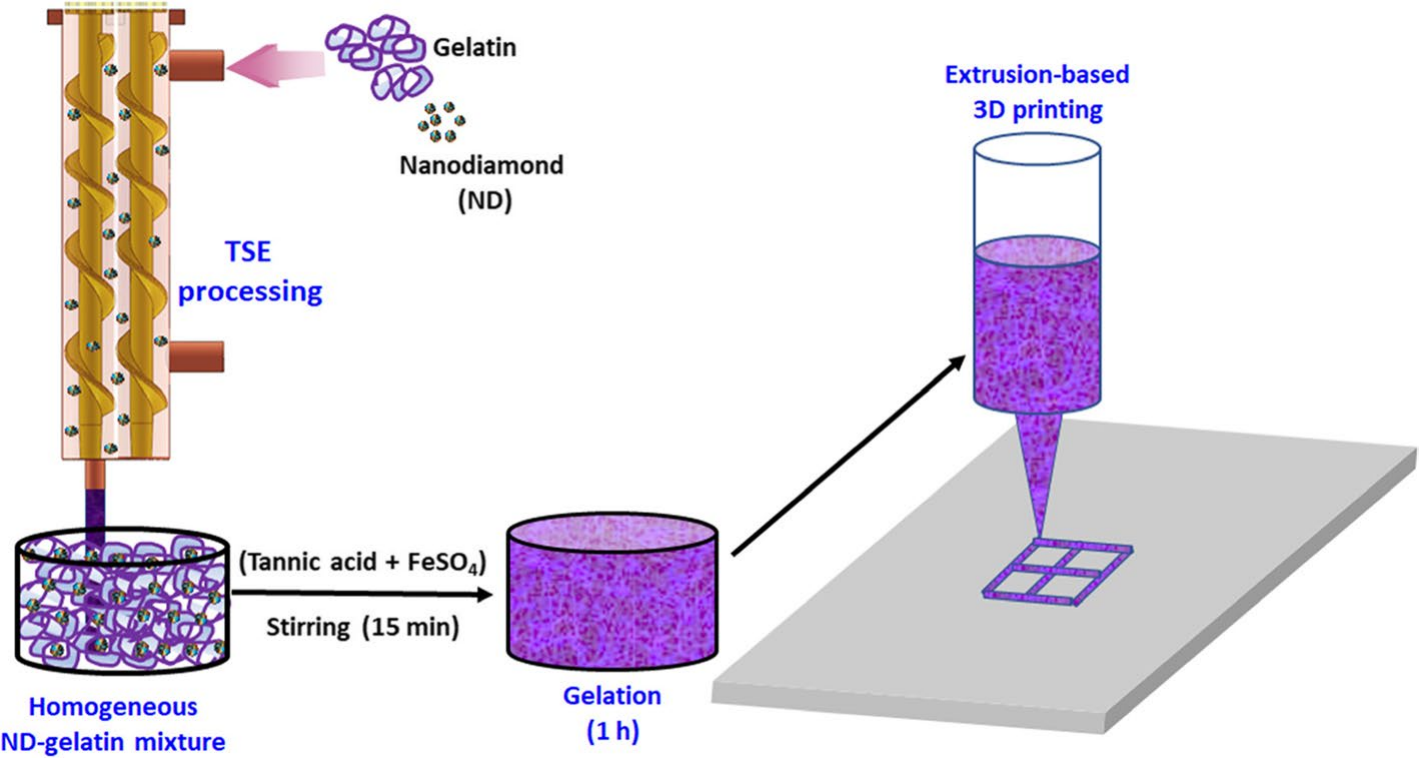
Scheme for the dispersion and subsequent 3D printing of the nanocomposite gelatin hydrogel. Homogeneous mixture of gelatin and NDs was obtained by the twin screw extruder (TSE), then TA and FS were added and stirred for 15 min. Finally, the ND-dispersed gelatin was kept undisturbed for 1 h gelation before extrusion-based 3D printing

For nanocomposite gel preparation, detonation nanodiamond (ND, Guangzhou Jiechuang Trading.co. Ltd. China, average particle size < 10 nm, agglomerate size of 20–50 nm) was added to the gelatin solution (2 g of gelatin in 8 ml of DI water). The advantages of using ND can be fully achieved if it is dispersed well in the polymer matrix [19]. Hence, for proper dispersion of ND in the gelatin matrix, the mixture was fed into the TSE head as described in our previous work [20] and extruded at 20 rpm. After this, TA solution and FS were added to the mixture, stirred for 15 min, and kept undisturbed for 1 h before use. Figure 1 shows the scheme for the nanocomposite gelatin gel synthesis and subsequent 3D printing.

Table 1 shows the optimized condition sample codes for different nanodiamond based hydrogels used in this paper. Other codes are explained in the figure caption.

**Table 1:** Sample codes for different nanodiamond contents in gelatin hydrogels

Composition	Nanodiamond (mg)	Code
Gelatin (2 g)-tannic acid (0.05 g)—Ferrous sulphate (0.02 g)-10 ml water	0	Gel-ND 0
	10	Gel-ND 1
	20	Gel-ND 2
	30	Gel-ND 3
	40	Gel-ND 4

### Characterization

#### Morphological and functional groups

The Structural morphology of the samples was determined by scanning electron microscopy (SEM, EVO 18 model from Carl Zeiss, Germany, Filament: Tungsten) and High-Resolution Transmission Electron Microscopy (HRTEM, 80 kV) from JEOL (JEM 2100, Japan). Selected area diffraction pattern (SAED) of ND was recorded using HRTEM. Other characterization techniques used in this study were XRD (Empyrean Malvern Panalytical diffractometer with MultiCore Optics, Cu Kα, λ = 1.54 Å), Fourier Transform Infra-Red Spectroscopy (FTIR 400–4000 cm^−1^ range, IR affinity series, Shimadzu, Japan), Particle Size Analyzer with zeta potential (Malvern Panalytical, Zetasizer Ver. 7.13), Rheometer (Anton Paar/Modular Compact Rheometer MCR 102, 37 ºC, Gap-1.025 mm, Measuring System- pp25) and Universal testing machine for compression test (INSTRON 3366, Speed-1 mm/min; dimensions of the cylindrical samples: 16 ± 0.1 mm diameter, 13 ± 0.2 mm height). ND (0.04 g) was dispersed in water with vortex shaking for 1 h and ultrasonic treatment of 15 min for particle size and zeta potential analysis. Dynamic Mechanical analysis (DMA) was carried out in the rheometer instrument in Torsional Mode (1% shear strain at 37 °C, frequency 1 – 10 Hz, cuboid samples: 10 mm × 5 mm × 3 mm).

#### 3D printing

A customized 3D bioprinting system (SeoulTech, Korea) was used for 3D printing [^21^]. The 3D lattice structure was designed using Solid Works software (Dassault Systems SolidWorks Corp, USA), and the G-codes for the STL files were generated using a slicing software (Simplify 3D version 4.0, USA), as described in our previous work [22]. The hydrogel (3 ml) was loaded inside the plastic syringe (5 ml, Musashi Engineering Inc., Korea) attached with a needle (22 gauge). The syringe needle was placed proximal to the stage with the substrate by adjusting the Z-axis (syringe holder), X and Y axis (stage) using the software. A printing speed of 150 mm/min with pressure of 50 kPa at a temperature 27 °C was used for 3D printing.

#### Cell culture studies

For cell culture experiments, an osteoblast cell line derived from mouse calvaria (passage 13, MC3T3-E1 cell line, Young Science Inc., Korea) was sub-cultured in a 24-well plate. α-Minimum essential media (MEM) (Sigma Aldrich, USA) containing 10% fetal bovine serum (Gibco Korea, Korea) and penicillin–streptomycin (100 unit/mL) was added to each well and incubated for 24 h with 5% CO_2_ at 37 °C. Then, 100 μL of media containing MC3T3 cells (0.5 × 10^6^/mL) was mixed with other sample solutions for different times and UV crosslinked before checking their live/dead ratio.

The different gel samples were prepared carefully under sterile conditions, and the gels were washed thoroughly with phosphate buffer saline (PBS) and medium to avoid any contamination. MTT (3-(4,5-dimethylthiazol-2-yl)-2,5-diphenyl tetrazolium bromide) assay was carried out using extraction protocol as described previously [23]. Dried gel samples (10 mg each) were soaked for 48 h in 1 mL of MEM cell culture medium and incubated at standard conditions to obtain the hydrogel extracts for the MTT assay. The MC3T3 (passage 13) cells were seeded on to the 96 well plate with 5000 cells/well (100 µL MEM/well). The medium was replaced with the extract liquid and incubated at standard cell culture conditions. The samples were assessed for cell viability at day 1, 2 and 3 using MTT assay. The in vitro cell culture study was performed up to 3 days in 24 well plate. The scaffolds were sterilized under UV radiation for 24 h and repeated washing with phosphate-buffered saline (PBS) and medium. The MC3T3 (passage 13) cells were seeded on the scaffolds (0.4 million/well) and the samples were observed on days 1, 2 and 3. Live/dead staining (LnD) was carried out using the LIVE/DEAD ® Viability/Cytotoxicity Kit (Invitrogen, USA). This kit combines green, fluorescent calcein-AM staining to show intracellular esterase activity with red-fluorescent ethidium homodimer-1 staining to confirm the loss of plasma membrane integrity. The images of the live and dead cells were taken after the addition of 1.2 μL of 2 mM ethidium homodimer-1 (EthD-1) and 0.3 μL of 4 mM calcein AM into 600 μL of PBS. After the addition of the two agents, the plate was incubated in the dark for 30 min. The live and dead cells were captured using different filters present in the fluorescence microscope (Leica DMLB, Germany) and merged using the LAS-X Leica microsystems software.

#### Statistical analysis

All images were analyzed using ImageJ software (NIH, USA) to estimate live/dead or cellular spreading on the gel surface. Statistical analysis was performed by one-way analysis of variance with 95% confidence (*p* ≤ 0.05) using the Tukey's Means Comparison Test with Origin 9.0 software.

## Results

### ***Optimization of gelatin/Tannic acid/FeSO***_***4***_*** for extrusion ability, disintegration, and swelling***

**Fig. 2:**
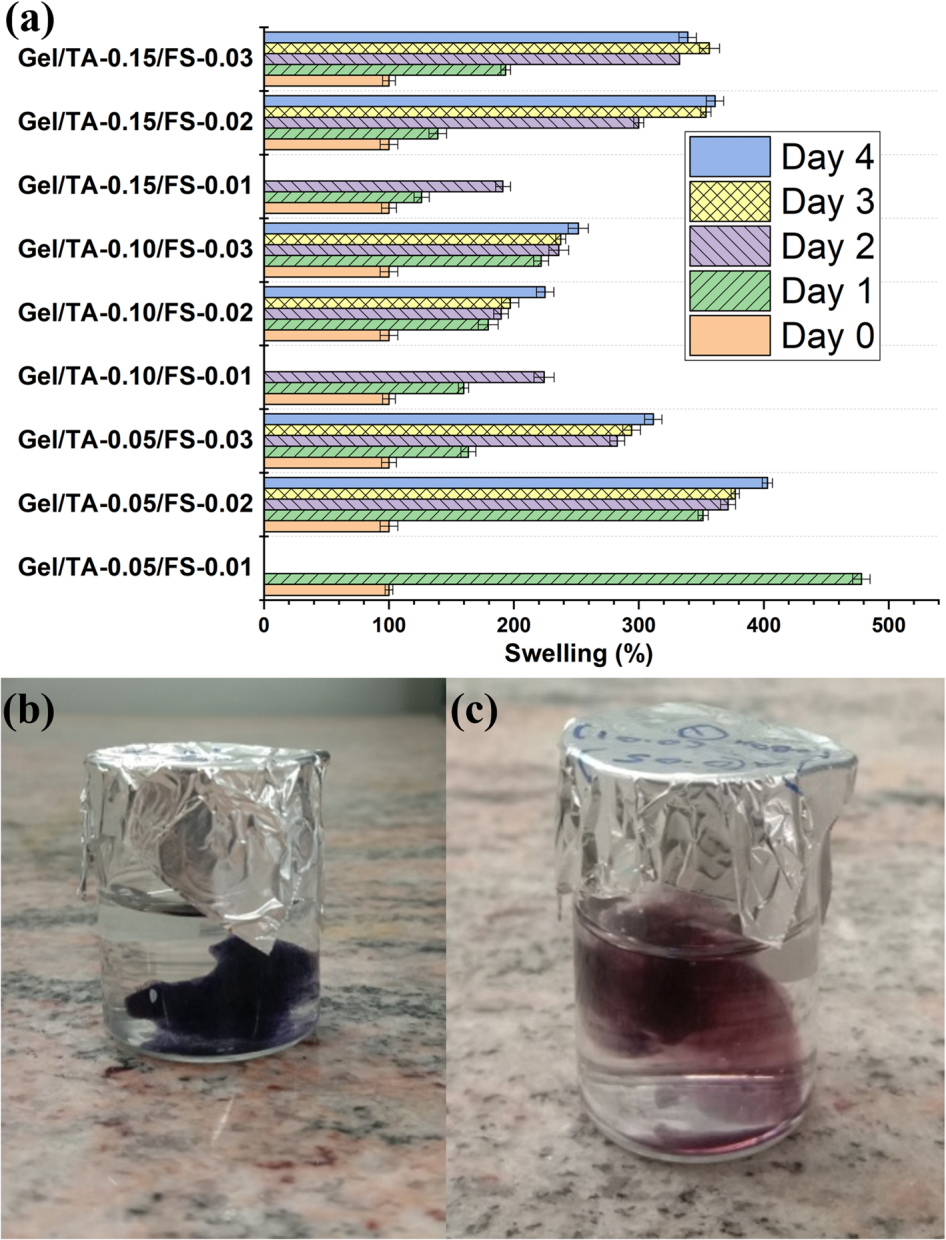
(**a**) Diameter swelling and disintegration of extruded different combinations (Gel: gelatin, TA: Tannic acid, FS: Ferrous sulphate; the amount (in g) of TA and FS mentioned in the samples, 2 g gelatin used in each case); Solubility test of extruded gel with higher water content (Gel/TA-0.1/FS-0.02) (**b**) at day 0, (**c**) at the end of day 2

Supplementary Table 1 shows the combinations used for optimization. The combinations were prepared, and the extrusion ability of the gels was checked. Among these combinations, gels with 0.05 g TA showed good extrusion ability. Samples with 0.1 g and 0.15 g TA were hard to extrude, so additional 5 ml of water was added to them. The prepared hydrogels were extruded with the help of a syringe and a needle. Supplementary Table 2 shows the extruded lines of different combination of gels. All these extruded hydrogels were subjected to swelling study by immersing the extruded gel in DI water at 37 °C. The thicknesses were recorded till Day 4 (Fig. 2). Samples with 10 mg FS (Gel/TA-0.05/FS-0.01) were not stable beyond 3 days. For Gel/TA-0.15/FS-0.03, slight reduction in mean value of swelling was observed at day 4 as it started disintegrating. The solubility test of extruded Gel/TA-0.1/FS-0.02 sample showed the evidence of disintegration after day 2 (Fig. 2b, c).

### Morphological characterization

**Fig. 3:**
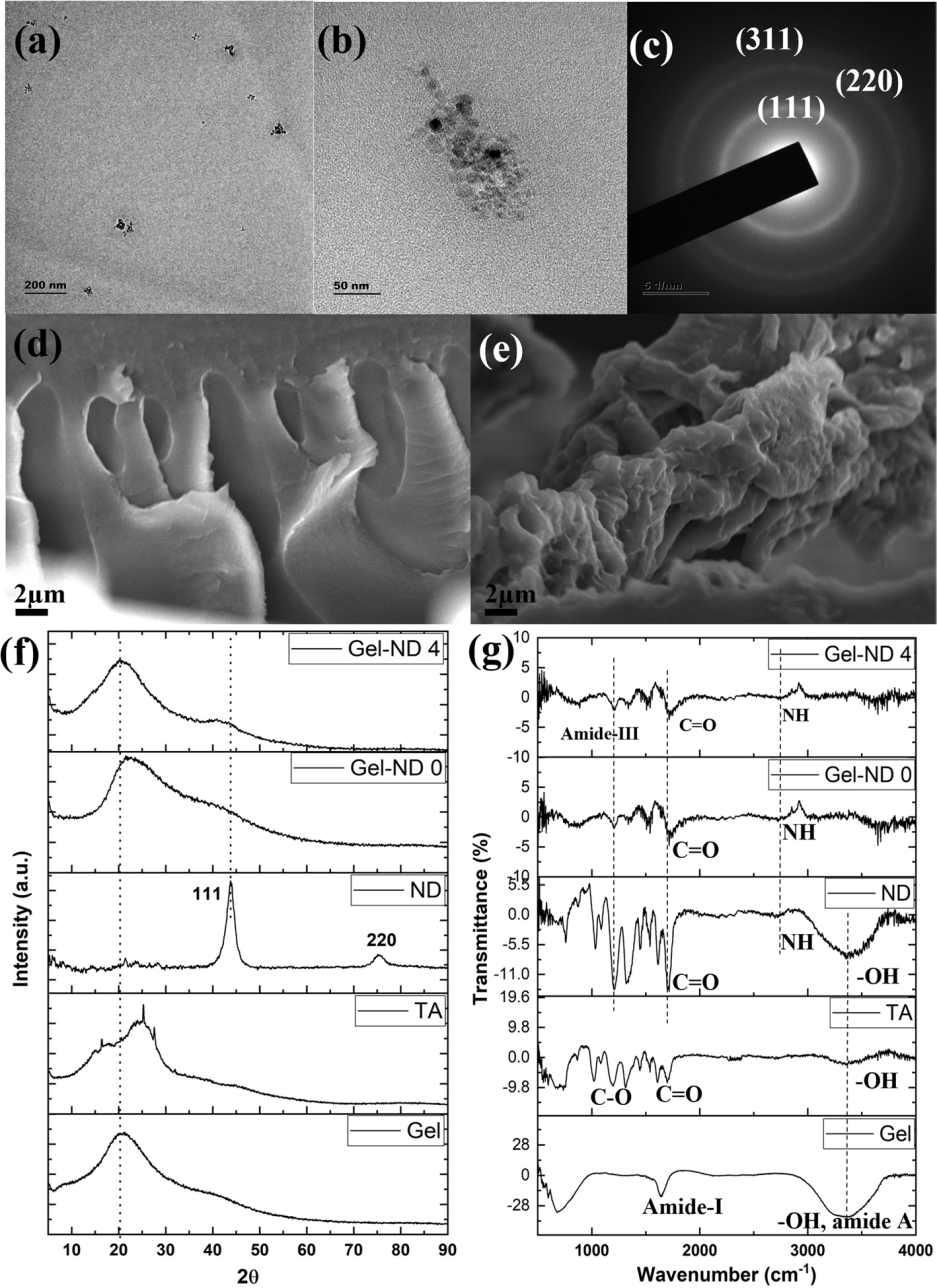
TEM image of (**a**) nanodiamond dispersed in gelatin, (**b**) high magnification showing cluster of ND, (**c**) SAED pattern with index; SEM images of dried cross section of (**d**) Gel-ND 0, (**e**) Gel-ND 4; (f) XRD and (**g**) FTIR spectra of different raw materials and synthesized samples. Gel: gelatin, TA: Tannic acid, ND: nanodiamond

Supplementary Fig. 1 shows the particle size distribution and zeta potential of ND dispersed in water with vortex shaking for 1 h and ultrasonic treatment of 15 min. An average size of 244 nm is recorded in particle size analysis which is much higher than the company reported one. Zeta potential was found to be + 9.16 mV indicating that the ND is meta-stable in water. Hence, for mixing of ND, our patented TSE head was used. The TEM Images were taken for the ND-dispersed gelatin at low accelerating voltage (80 kV) as gelatin degrades at high voltage (Fig. 3a). Bigger cluster was also observed as shown in Fig. 3b. The crystal planes of ND were found in SAED pattern (Fig. 3 c). The indexing of SAED pattern confirmed the crystal planes of ND at (111), (220), (311) [24]. SEM images revealed a porous structure of the crosslinked gels with and without ND (Fig. 3d, e). The XRD peaks were used to determine the crystallography of the material (Fig. 3f). XRD of gelatin showed a wide amorphous diffraction peak at 2θ = 20.1º [25]. TA gives diffraction peak at 2θ = 25º proving its amorphous nature. Addition of TA changed the order of gelatin molecules due to the large number of phenolic hydroxyl groups disrupting the hydrogen bonds of gelatin [26]. Nanodiamond peaks were observed at θ = 41º and 75º corresponds to (111) and (220) planes of the crystal structure, respectively. After ND inclusion, a small hump was observed around θ = 41º for Gel-ND 4 sample indicating the presence of ND in the structure. FTIR of gelatin showed a band at 3436 cm^−1^ due to the presence of water (O–H stretching) and amide A (Fig. 3 g). Peak at 1637 cm^−1^ confirmed amide-I in gelatin [25]. FTIR of TA showed a broad absorption band at 3600 cm^−1^–3000 cm^−1^ corresponds to the -OH groups H-bond. The presence of aromatic esters in TA was evident through ester and carbonyl groups C = O stretching (1820 – 1670 cm^−1^) and peaks at 1300 – 1000 cm^−1^ for alcohol, ether, and ester C-O vibration [27]. FTIR spectrum of ND confirmed that the ND used in this study was the detonation ND [28]. Band at 3400 cm^−1^ indicated O–H symmetric stretching while N–H and C = O stretching were observed at 2750 cm^−1^ and 1750 cm^−1^, respectively. Other peaks were identified as C-O bending, hydroxyl (1026 cm^−1^), amide III (1210, 1320 cm^−1^) and C-N stretching, amide II (1535 cm^−1^). FTIR spectrum of Gel-ND 4 showed amide III peak (1203 cm^−1^) and C = O symmetric stretching (1750 cm^−1^). The N–H stretching peak was observed at 2750 cm^−1^.

### Rheology and compression

**Fig. 4:**
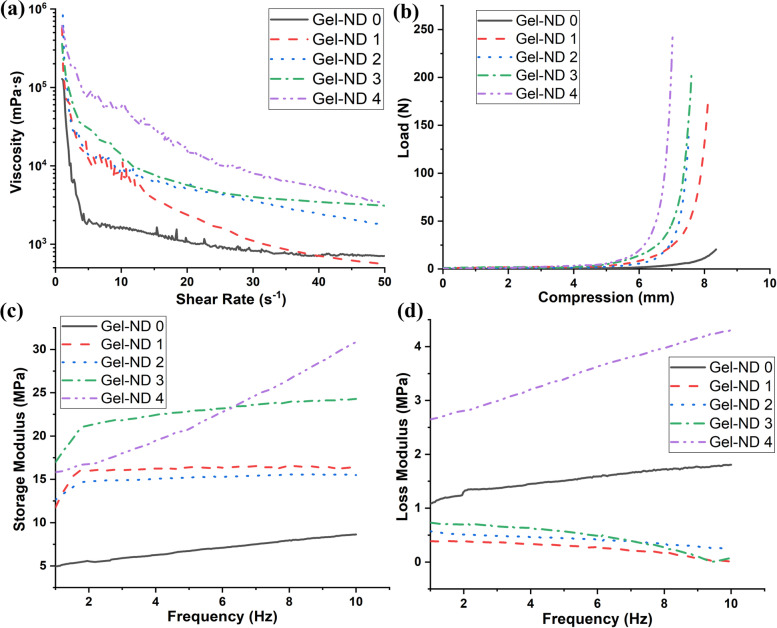
Rheological properties of ND-incorporated gelatin hydrogel; (**a**) parallel plate rheology of different gels, (**b**) compressive curves for cylindrical gel samples, dynamic mechanical properties of gels, (**c**) storage modulus, and (**d**) loss modulus

The rheology of these gels revealed the shear-thinning property of all the synthesized hydrogels (Fig. 4a). With the increase in ND content, the viscosity of the gels increased making it more difficult to extrude. The compressive test also indicated strength improvement and less extension with the addition of ND (Fig. 4b). The dynamic mechanical analysis (in torsion mode) revealed almost three times improvement in storage or elastic modulus with ND reinforcement at 1% shear strain (Fig. 4c). Figure 4 (d) shows the viscous or loss modulus of the hydrogels. In dynamic mechanical analysis, gelatin hydrogel (Gel-ND 0) and Gel-ND 4 showed similar strain-hardening effect with frequency. Other ND-reinforced hydrogels showed a higher increase up to 2 Hz and then the increase was not so sharp. Gel-ND 3 showed higher storage modulus than Gel-ND 4 till 6.5 Hz frequency, though their initial storage moduli were almost same.

### Extrusion ability, swelling and disintegration

**Fig. 5:**
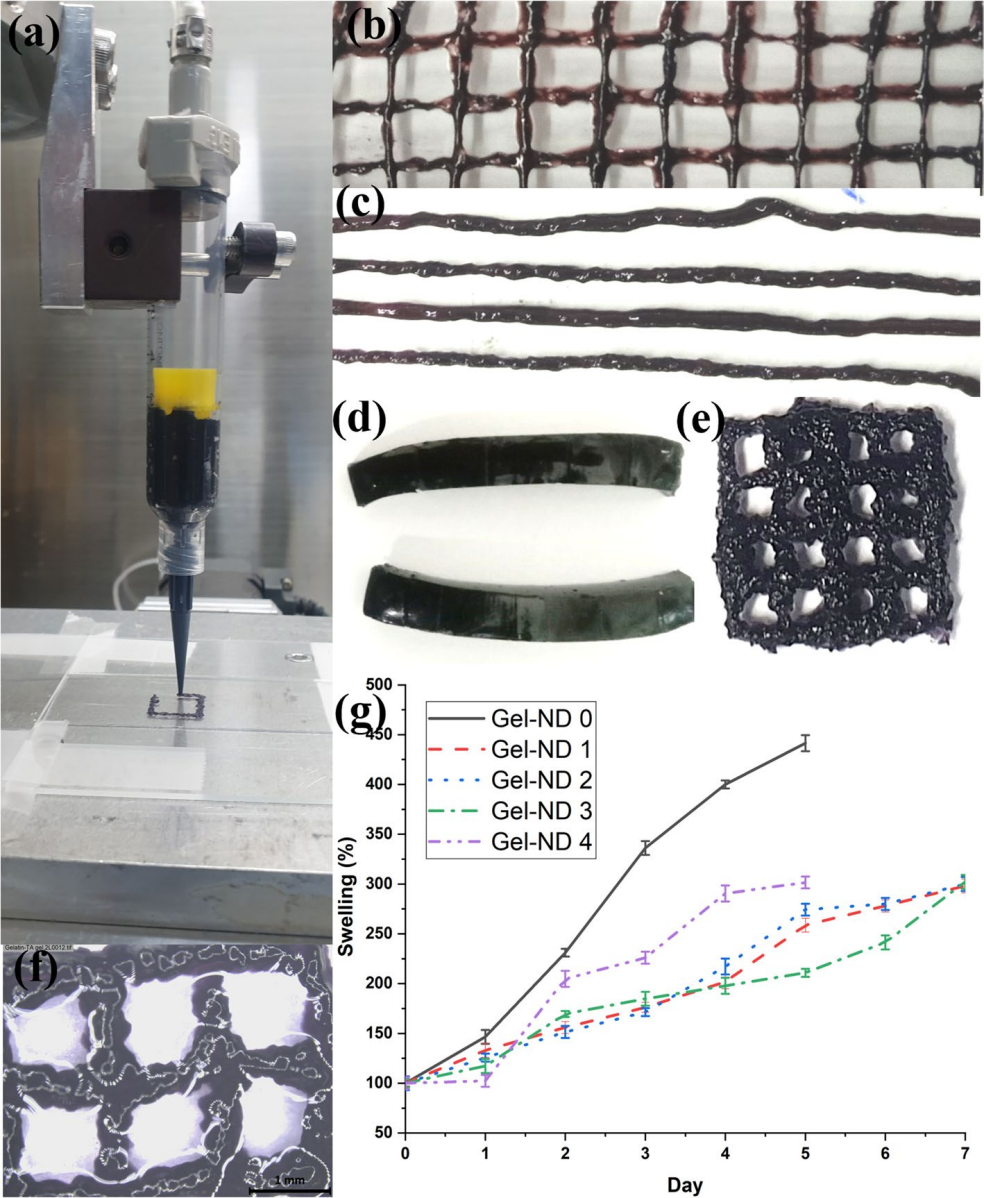
(**a**) 3D printing of Gel/TA/FS gel, and its behaviours: (**b**) grid like extrusion (approx. spacing 10 mm, thickness 1 mm), (**c**) extruded lines through hand, (**d**) cuboid shape gels, (**e**) 3D bioprinted gel grid, (**f**) microscopic image of 3D bioprinted grid, (**g**) swelling in DI water

Figure 5 demonstrates the extrusion ability of the prepared hydrogels. The gels were loaded in the 3D printer syringe and grid-like structures were created (Fig. 5a). Figure 5 (b) and (c) show the extruded lines using syringe and 22-gauge needle by hand. Hand pressure was found to be sufficient for a good continuous flow. Figure 5 (d) shows the cuboid samples prepared for dynamic mechanical analysis. The 3D printed gel (4 layers) and its microscopic image are shown in Fig. 5 (e) and (f), respectively. The disintegration of the structure and its swelling were observed for different gels by studying the change in thickness till day 7 (Fig. 5 g). The gels swelled for about 5 days and then started disintegrating in water.

### In vitro cytocompatibility analysis

**Fig. 6:**
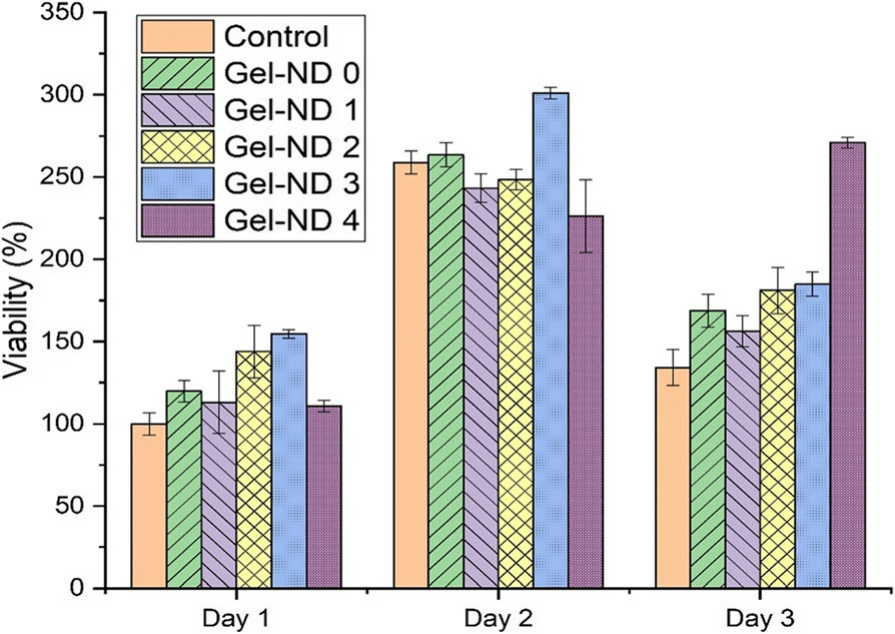
MTT assay for cytotoxicity of different gels with MC3T3 (passage 13) cells

**Fig. 7:**
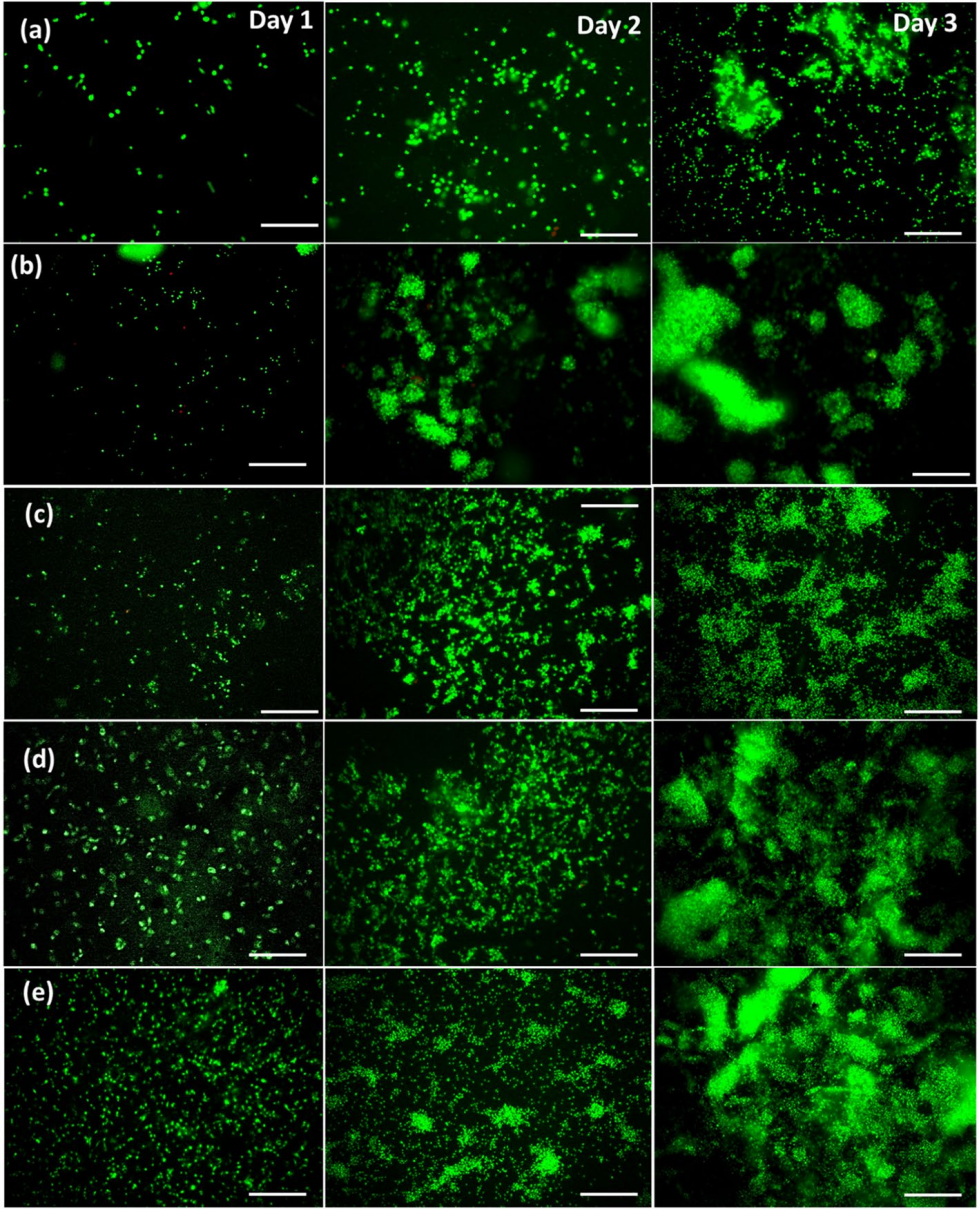
Live/dead stained images of different nanocomposite hydrogels in Day 1, 2 and 3 MC3T3 (passage 13) cells, (**a**) Gel-ND 0, (**b**) Gel-ND 1, (**c**) Gel-ND 2, (**d**) Gel-ND 3, (**e**) Gel-ND 4. (Scale bar 250 µm)

The response and cytotoxicity of various nanocomposite hydrogels on MC3T3 cells were investigated by the MTT assay till day 3 (Fig. 6). Day 1 control was considered as 100%. All the sample results were more than 100% viability indicating good cytocompatibility of the hydrogels used. The live/dead stained images of the samples for day 1, 2 and 3 showed the cell growth and proliferation in the nanocomposite gel matrix (Fig. 7). Number of dead cells (red) was found to be negligible compared to the live cells (green).

## Discussion

Hydrogels can be formulated in a variety of physical forms and thus easy to use in clinical practice and medicine for a wide range of applications, including tissue engineering and regenerative medicine. After injecting or transplanting them inside body, they can maintain their structure and slowly degrade over time resulting in controlled release. Cell encapsulated gelatin based injectable hydrogels are in clinical trial phase II stage under U.S. Food and Drug Administration (FDA) [29]. Hence, it is essential to explore the possibilities to get biocompatible crosslinked gelatin based injectable hydrogels for future use. In this work, extrudable gelatin-based hydrogel with moderate stability is targeted and its mechanical and biological properties are improved using detonation ND. A natural crosslinker TA and essential body mineral FS are used for crosslinking the gel to achieve good biocompatibility. Gelatin contains amino functional groups like lysine, arginine, and histidine. These can react with TA through its phenolic sites under alkaline conditions to form cross-linked network using covalent C-N bonds. The carboxyl and phenolic hydroxyl groups of TA can also form hydrogen bonding with the amino groups of gelatin chains. The extent of these reactions and interactions in gelatin/TA systems play an important role in determining consequent hydrogel properties [6].

Samples with higher TA amount of 0.1 g and 0.15 g were found to be difficult to extrude. The addition of excess water for successful extrusion may reduce the FeSO_4_ (FS) efficiency for crosslinking and hence, the samples with 0.1 g and 0.15 g TA disintegrate faster. Looking into all combinations used for extrusion and subsequent disintegration and swelling study, 0.05 g TA and 0.02 g FS were selected for studies with ND. This combination contains low amount of TA and FS with good extrusion ability and moderate stability over 4 days. This can help slow release of biomolecules or live cells to the target site.

The use of detonation ND in this study is confirmed through TEM, XRD and FTIR. Detonation ND is researched for different biomedical applications in its nano-biocomposite form. These include bone tissue engineering, chemotherapeutic drug delivery and wound healing. It is possible to have large scale production of ND with narrow size distribution through detonation technique. However, the dispersion of ND agglomerates in water is very difficult due to its high surface area and large number of surface functional groups, as mentioned in the introduction. Particle size data reported agglomerated ND even after extensive mixing process with ultrasonic treatment. Hence, shear mixing was introduced using our patented TSE head. Figure 3 (a) reveals that the ND is well distributed in the gelatin matrix and most of them are less than 50 nm clusters. It is possible to employ still higher shear rate using our developed TSE head to disperse the bigger clusters or agglomerates, however, further shear rate can damage the gelatin molecular chain, leading to lower molecular weight and lower viscosity in gel.

For good extrusion printing, shear thinning property of hydrogels is very important to retain the post-printing fidelity of the printed gel scaffolds. The high surface area of ND and its ability to participate in the crosslinking of gelatin/TA system through its surface functional groups result in viscosity increase with its concentration in gel matrix. Though ND increases the viscosity of the nanocomposite hydrogels, the shear thinning property of the hydrogels remains same with the increase in shear rate. The compressive strength improvement compared to pure gelatin gel is very high (almost 10 times) owing to good dispersion of ND in the gel matrix and its interaction with the Gelatin/TA system through its surface charges. The surface charges of detonation ND help to build strong interfacial bond between the nano reinforcement and matrix. This helps to successfully transfer the load on the nanocomposite hydrogel to the ultra-strong ND. Thus, ND improves the load bearing capacity of the gel, though the extension is compromised due to better crosslinked structure. This is also observed in the case of dynamic mechanical properties. With higher amount of ND in the structure, it is more difficult to disperse it uniformly which can result in loss in elastic property and increase in viscous property. After addition of NDs, the viscous component reduces for all samples except Gel-ND 4 compared to gelatin. This indicates that the amount of agglomeration or inhomogeneity in gel structure is higher in the case of Gel-ND 4. The shear mixing conditions used in this work is not sufficient for higher loading of ND. Higher shear rate system should be used in such cases. However, that may result in destruction of polymer chain and reducing its molecular weight to make it inefficient for gel formation, especially in 3D printing.

The gel extrusion experiments revealed that this gel can be extruded using hand or 3D printer with reasonable resolution for subsequent uses. The dried gels have pores in their structures, which will help the cells to attach, grow, and proliferate on the hydrogel scaffold. Cell-loaded gels can also be printed (3D bioprinting) using these gels as the process does not require any harsh physical or chemical treatment during the gelation and subsequent 3D printing. This makes it suitable for such cell-encapsulated 3D bioprinting applications in biomedical and tissue engineering fields. Other than extrusion ability, the structure disintegration and swelling behaviours are also important to understand the possible application areas of the hydrogel. To validate these properties, nanocomposite gelatin hydrogels were extruded (line) and swelling test was carried by immersing the extruded (line) samples in DI water at 37 °C. It is evident from the Fig. 5 g that the incorporation of ND improves the stability of the gelatin hydrogel. Pure Gel/TA/FS (Gel-ND 0) cannot retain its structure beyond Day 5, while ND-based samples are holding their shape till day 7. However, at higher ND loading of 40 mg, the agglomeration problem may result in lowering the stability of gelatin hydrogel. As shown in supplementary Fig. 2, Gel-ND 3 sample shows good stability in water. The percentage swelling calculated for each sample indicates that ND reduced the swelling of gelatin hydrogel significantly (from 341% (in day 5) in Gel-ND 0 to ~ 200% for the ND-based samples. This will help to retain the structure with less deformation for the moderate time duration till the cells grow and cover the entire bioprinted region.

In MTT assay, yellow tetrazolium salt is reduced to purple formazan crystals by dehydrogenase enzymes from the metabolically active cells. The amount of purple formazan crystals is proportional to the number of viable cells. Hence, this is used to assess the in vitro cytocompatibility and increase in cellular proliferation of different nanocomposite hydrogels. For cytotoxicity assessment through MTT assay, more than 70% cell viability is considered as nontoxic [30]. Cell viability measured using MTT assay indicates that all the gels are nontoxic and cell supportive. The viability is found to be more than 100% in all the cases employed in this study (Fig. 6). The live and dead staining carried out on all the samples at Day 1, 2 and 3 indicates increase in number of cells with days. ND has positive influence on the attachment, growth, and proliferation of the cells [31]. In line with earlier observations, the increase in ND enhances the growth and proliferation of the cells in this study also (Fig. 7). Among the samples, Gel-ND 4 with highest amount of ND employed in this study (Fig. 7-e) showed the best result with almost four times more cells in the scanned areas compared to the pure gel. All ND-based samples have higher cell growth and proliferation compared to the pure gelatin hydrogel. Hence, ND contributed significantly for the growth of the cells in these gels. This nanocomposite hydrogel can be successfully used to provide a suitable microenvironment for the adhesion, growth, and proliferation of the encapsulated cells. The controlled degradation and higher mechanical properties of this injectable and printable hydrogel make it a promising candidate to support the cells for a considerable time in the controlled microenvironment such that the cells can adjust the target site environment and gradually replace to form neo tissue.

## Conclusion

Gelatin based hydrogels are attractive in biomedical applications like tissue engineering. The cell encapsulated injectable hydrogels based on gelatin is in clinical trial stage. Hence, considering the advancements in 3D bioprinting, the naturally crosslinked cyto-compatible gelatin is an attractive choice for the researchers working to achieve better extrusion and structural stability in such hydrogels. This study highlights the tannic acid and ferrous sulphate combination for safe use in crosslinking to get extrudable and printable gelatin-based hydrogels. The process does not have any complicated chemical cross-linking agents and synthesis steps. The mixture of gelatin and ND obtained by the TSE shows good extrusion ability with the help of TA and FS molecules for cross-linking with gelatin hydrogel matrix and has moderate post-printing stability for 4 days. This can help slow release of biomolecules or live cells to the target site. ND improves the post-printing stability of gelatin hydrogels with the help of TA and FS. It also improves the mechanical performances of gelatin hydrogels depending on its concentrations. The gelatin hydrogel shows excellent cell compatibility, which is important in its applications as scaffolds for tissue regeneration such as skin wound dressing. These materials can be tested for improved post-printing stability with higher dosing of crosslinker as well as for extrusion-based 3D bioprinting in future. These ND-incorporated hydrogels can be employed for modulation of the 3D bioprinting scaffold in tissue engineering and biomedical applications such as skin wound dressing for its degradations, printability, post-printing fidelity and mechanical properties.

## Supplementary Information

**Additional file1:** **Supplementary Table 1.** Different combination of components used for optimization. **Supplementary Table 2.** Extrusion images of different gel samples (Gel: gelatin, TA: Tannic acid, FS: Ferrous sulfate; the amount (in g) of TA and FS mentioned in the samples, 2 g gelatin used in each case). **Supplementary Figure 1.** ND in water using dynamic light scattering (DLS) technique, (a) particle size distribution, (b) zeta protentional. **Supplementary Figure 2.** Solubility test for nanocomposite gel.

## Data Availability

Available on request.
