# Supplementary material for: Nanodiamond enhanced mechanical and biological properties of extrudable gelatin hydrogel cross-linked with tannic acid and ferrous sulphate
Source: Biomater Res. 2022 Jul 30;26:37. doi: 10.1186/s40824-022-00285-3 (PMC9338610; doi:10.1186/s40824-022-00285-3)
Supplement: Supplementary file 1 — Additional file1: Supplementary Table 1. Different combination of components used for optimization. Supplementary Table 2. Extrusion images of different gel samples (Gel: gelatin, TA: Tannic acid, FS: Ferrous sulfate; the amount (in g) of TA and FS mentioned in the samples, 2 g gelatin used in each case). Supplementary Figure 1. ND in water using dynamic light scattering (DLS) technique, (a) particle size distribution, (b) zeta protentional. Supplementary Figure 2. Solubility test for nanocomposite gel. [file 40824_2022_285_MOESM1_ESM.docx]

**<Supplementary Information>**

**Nanodiamond enhanced mechanical and biological properties of extrudable gelatin hydrogel cross-linked with tannic acid and ferrous sulphate**

Amitava Bhattacharyya ^1, 2, 3^, V. N. Karthikai Priya ^3^, Ji-hyun Kim ^1^, Mst Rita Khatun ^1^, R. Nagarajan ^3^, Insup Noh ^1, 2*^

^a^ Department of Chemical and Biomolecular Engineering, Seoul National University of Science and Technology, Seoul 01811, Republic of Korea

^b^ Convergence Institute of Biomedical Engineering and Biomaterials, Seoul National University of Science and Technology, Seoul 01811, Republic of Korea

^c^ Functional, Innovative and Smart Textiles, PSG Institute of Advanced Studies, Coimbatore 641004, India **insup@seoultech.ac.kr*

**Supporting data file**

Table 1. Different combination of components used for optimization.

| Gelatin (g) | Tannic acid (g) in 2 ml water | FeSO_4_ (g) |
| --- | --- | --- |
| 2 in 8 ml water | 0.05 | 0.01 |
|  |  | 0.02 |
|  |  | 0.03 |
|  | 0.1 | 0.01 |
|  |  | 0.02 |
|  |  | 0.03 |
|  | 0.15 | 0.01 |
|  |  | 0.02 |
|  |  | 0.03 |

Table 2. Extrusion images of different gel samples (Gel: gelatin, TA: Tannic acid, FS: Ferrous sulfate; the amount (in g) of TA and FS mentioned in the samples, 2 g gelatin used in each case)

| **Sample** | **Image** |
| --- | --- |
| Gel/TA-0.1/FS-0.01 | 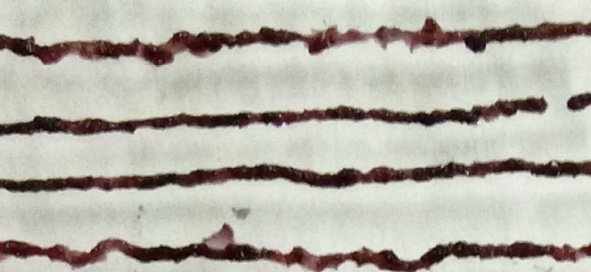 |
| Gel/TA-0.1/FS-0.02 | 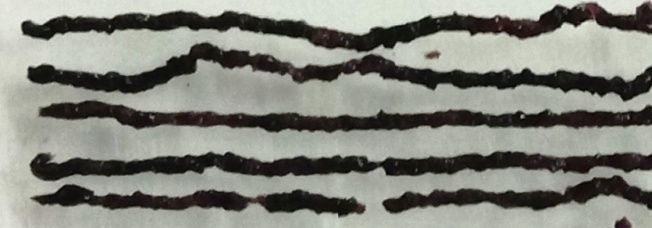 |
| Gel/TA-0.1/FS-0.03 | 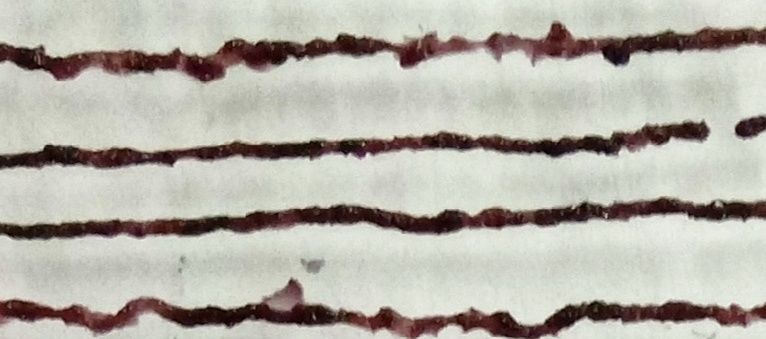 |
|  |  |
| Gel/TA-0.05/FS-0.01 | 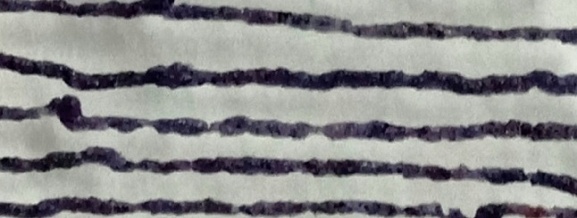 |
| Gel/TA-0.05/FS-0.02 | 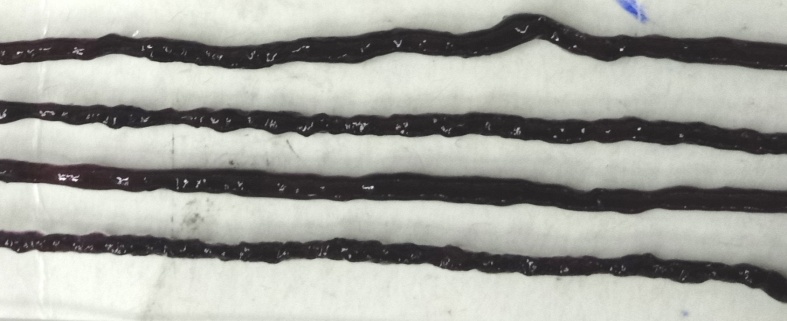 |
| Gel/TA-0.05/FS-0.03 | 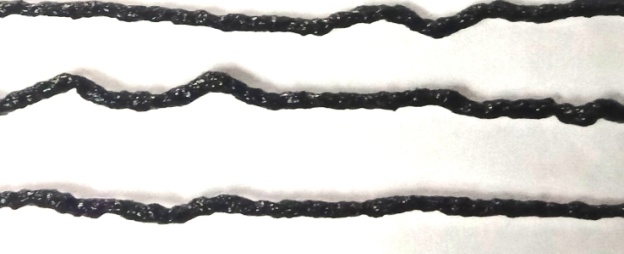 |
| Gel/TA-0.15/FS-0.01 | 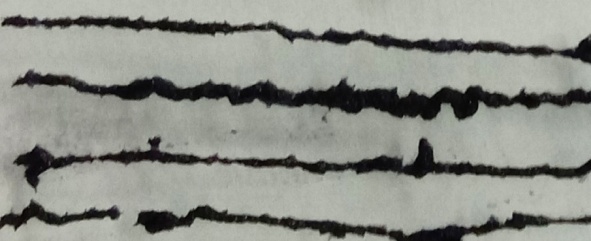 |


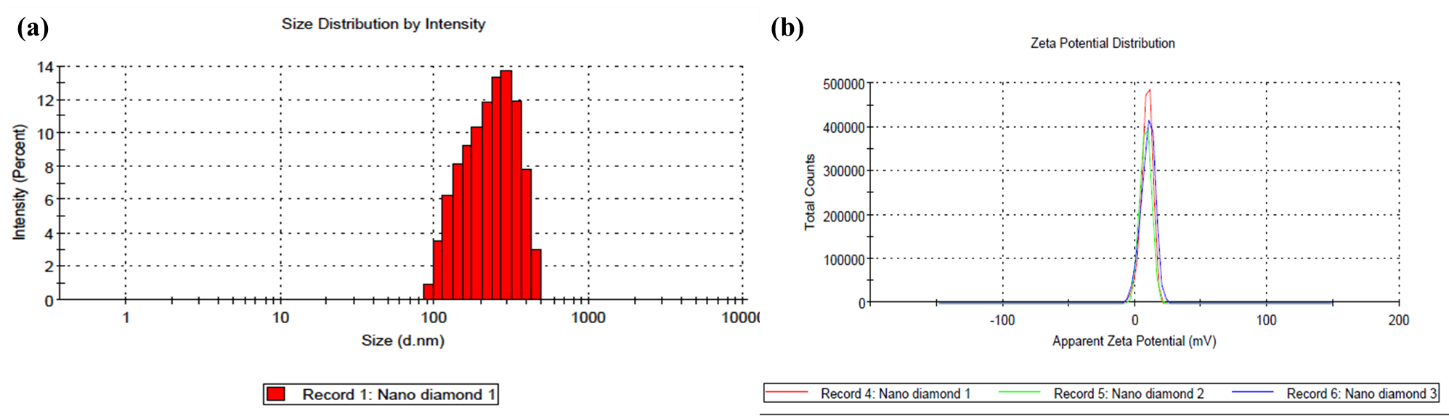


Figure 1: ND in water using dynamic light scattering (DLS) technique, (a) particle size distribution, (b) zeta protentional


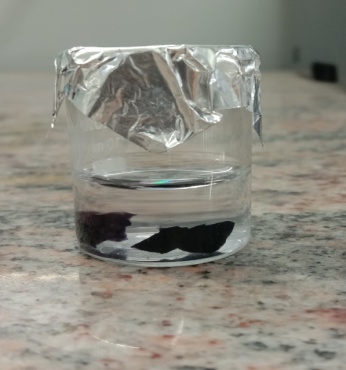

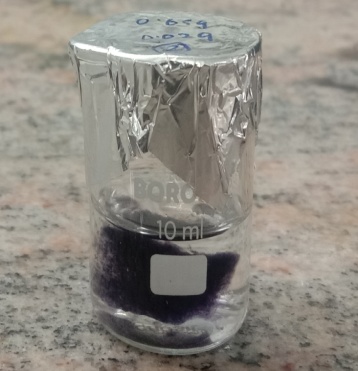


Gel-ND 3 kept for solubility Gel-ND 3 insoluble after 6 days

Figure 2: Solubility test for nanocomposite gel
